# Para-Hamiltonian form for General Autonomous ODE Systems: Introductory Results

**DOI:** 10.3390/e24030338

**Published:** 2022-02-26

**Authors:** Artur Kobus, Jan L. Cieśliński

**Affiliations:** Faculty of Physics, University of Bialystok, ul. Ciołkowskiego 1L, 15-245 Białystok, Poland; j.cieslinski@uwb.edu.pl

**Keywords:** Hamiltonian dynamics, phase space geometry, dissipative systems, conservation laws, M-systems of ecology, van der Pol oscillator, Brusselator, Lotka–Volterra model, Robertson reactions, Nosé–Hoover system, 37N05, 70H33, 34A34, 53Z05

## Abstract

We propose a new tool to deal with autonomous ODE systems for which the solution to the Hamiltonian inverse problem is not available in the usual, classical sense. Our approach allows a class of formally conserved quantities to be constructed for dynamical systems showing dissipative behavior and other, more general, phenomena. The only ingredients of this new framework are Hamiltonian geometric mechanics (to sustain certain desirable properties) and the direct reformulation of the notion of the derivative along the phase curve. This seemingly odd and inconsistent marriage of apparently remote ideas leads to the existence of the generator of motion for every autonomous ODE system. Having constructed the generator, we obtained the Lie invariance of the symplectic form ω for free. Various examples are presented, ranging from mathematics, classical mechanics, and thermodynamics, to chemical kinetics and population dynamics in biology. Applications of these ideas to geometric integration techniques of numerical analysis are suggested.

## 1. Introduction

Physics clearly distinguishes systems with conservation laws obeyed, as this simplifies the treatment of physical input given by initial or boundary conditions and a formal description in terms of admissible mathematical tools. Hamiltonian systems, which are model examples of how conserved quantities fit into physics, are very convenient since their nice formal features and associated structure provided by symplectic geometry additionally reveal several hidden links between objects engaged in the theory [1]. Nonetheless, often, the analytic methods are not sufficient, as we need to ponder the possibility of the numerical solution of the problem under consideration or even some trickier purely formal methods, as asymptotic expansion in the vicinity of singularities [2].

This article proposes some form of these “trickier methods” that could be applied to numerical algorithms, as they offer a weak form of the conservation laws (we mean here a conserved charge without the corresponding classical conservation law.

Numerical integration algorithms range from general-purpose classic Runge–Kutta and multi-step schemes [3] to more specific, geometric methods [4]. The latter admit of course better qualitative behavior (e.g., long time stability [5]), while the former usually are of higher order with less-flexible adaptation possibilities when it comes to the change of the simulation circumstances (e.g., the scheme fails to maintain high accuracy when the simulation lasts too long). Hence, geometric methods show some superiority, when we go to the extremes of bending the simulation parameters.

We found the main motivation of the undertaken research in the geometric numerical treatment of ODEs known as Geometric Numerical Integration (GNI) (see, e.g., [4,6,7,8,9]), although in this paper, we confine ourselves mainly to the construction of the proper continuous counterpart of the discrete framework needed for the treatment of general autonomous, non-conservative systems.

As is well known, geometric methods demand some kind of in-built qualitative structure of the considered problem to be preserved by the scheme. A good example of such a structure, and maybe the simplest one, is a single conserved quantity. This case can be reduced to the usage of a certain discrete gradient algorithm; see, e.g., [10,11,12,13]. The last reference here is an excellent example of accessing the second invariant quantity through discrete gradients. Such a numerical treatment was indeed applied to systems with first integrals and Lyapunov functions, but for arbitrary systems, it generally ceased to function properly because of a lack of a structural property guiding the evolution of the system. Although several frameworks of interest have been proposed for the qualitative description of such problems (see, e.g., [14,15,16,17,18]), none of these provides a simple and immediate way to gain access to the conserved quantity, helping directly in the performance of a specially crafted numerical algorithm.

In this paper, we show that Hamiltonian systems are indeed ubiquitous when we consider some consequences of the theorem on the existence and uniqueness of solutions to ordinary differential equations (ODEs) [19,20,21] and seek for the structure described above even if it is apparently absent. This approach leads to an abundance of so-called effective integrals of motion.

To begin with, let us consider the simple Initial-Value Problem (IVP) for an autonomous ODE:

(1) $$\dot{x}=f\left(x\right),\;x\left(t_{0}\right)=x_{0},$$

given on some open domain $D\subset R^{d}$ with $f:D\to R^{d}$; obviously, an overset dot stands for a shortcoming denoting time derivation.

Given $f\left(x\right)\in C^{0}\left(D\right)$, the existence and continuity of the solutions are guaranteed. The assumption of $f\left(x\right)\in C^{r}\left(D\right),r⩾1$ ensures the uniqueness of the solution and its respective differentiability properties [1,19,21].

McLachlan et al. [12,22] showed that, in a neighborhood of a non-degenerate fixed point of a dynamical system, the existence of the first integral of (1) is equivalent to the existence and boundedness of skew-symmetric matrix B(x) such that:

(2) f(x)=B(x)∇H(x),

where H(x) denotes the mentioned integral. Note that *B* is not determined uniquely, since we can add any solution of the homogeneous equation 0=B∇H to the particular solution:

(3) $$S^{\left(P\right)}=\frac{1}{{\left|\nabla H\right|}^{2}}f\wedge \nabla H$$

The exterior product could be defined as:

(4) $$v\wedge w:=\sum_{i,j}\left(v_{i}w_{j}-v_{j}w_{i}\right),\;i,j=1,2,\ldots ,d,$$

being explicitly anti-symmetric (note that in $R^{3}$, the structure provided by such a product is equivalent to the one given by the usual cross-product). The Formula (3) explicitly demands |∇H|≠0. However, if we are already given the problem in the form of (2), the assumption of ∇H≠0 is redundant.

This article is meant to serve to extend the classical Hamiltonian approach to non-conservative systems, therefore unifying the perspective of gradient discretization for all autonomous ODEs and their study, as this has its origin in [22]. The approach dedicated to even more general systems (non-autonomous, optimal control, stochastic) is still under development.

In Section 2, we provide an overview of the subject of geometric mechanics (for the notation and basic terminology, check [1,23]), Section 3 serves in the same way to refresh the basic information on the biological population and chemical reaction dynamics. Section 4 introduces the main ideas of the paper, emphasizing direct overuse of the existence and uniqueness theorem for ODE systems. Section 5 presents versatile examples of the application of the introduced formalism, and Section 6 gives concluding remarks.

## 2. Hamiltonian Mechanics

If B(x) in (2) obeys the Jacobi identity, we can term the system Poisson or Hamiltonian (non-canonical). Since Poisson structure matrix *B* is always of an even rank [24], odd-dimensional systems are necessarily degenerate (some even-dimensional ones also are; this implies the existence of Casimir functions) [25,26].

On the chosen symplectic leaf with prescribed Clebsch coordinates, treated as symplectic manifold $\left(T^{*}M,\omega \right)$, we have:

(5) $$\omega =dx^{i}\wedge dp_{i}$$

(the summation convention assumed throughout the paper) the existence of which is equivalent to the non-degenerate canonical Poisson bracket given by the Poisson bi-vector:

(6) $$\pi =-\omega^{-1}:\overset{2}{⋀}T^{*}M\to R.$$

where:

(7) π(df,dg)={f,g},

and the $f,g\in F(T^{*}M)$ Lie algebra of functions defined on the phase space.

This also gives rise to the first-order differential operator:

(8) $$X_{H}\left(\cdot \right)=\pi \left(\cdot ,dH\right)$$

known as a Hamiltonian vector field.

Now, we call it the the first integral generator of motion if:

(9) $$\dot{x}=\left\{x,H\right\}=X_{H}\left(x\right)$$

understood componentwise. In other words:

(10) $${\dot{x}}^{i}=\frac{\partial H}{\partial p_{i}}\;,\;{\dot{p}}_{i}=-\frac{\partial H}{\partial x^{i}}\;.$$

or simply:

(11) $$X_{H}\rfloor \omega =dH,$$

denoting by ⌋ the substitution of a vector field into the form ω (contraction).

Note that the flow of the Hamiltonian vector field preserves the canonical symplectic form on the phase space, which is clearly given by the proper Lie–Ślebodziński derivative:

(12) $$L_{X_{H}}\left(\omega \right)=X_{H}\rfloor d\omega +d\left(X_{H}\right\rfloor \omega )=d\left(dH\right)=0,$$

by the closedness of the symplectic form and the nilpotency of exterior derivative *d*.

The basic hydrodynamic interpretation of the canonical formalism is also of some value. Let us consider a phase fluid of many systems with various initial conditions, moving on the phase space [27]. The velocity field of the fluid is clearly given by the Hamiltonian vector field. Note that since $H\in C^{2}\left(D\right)$, we have divv=0.

It surely obeys the continuity law:

(13) $$\frac{\partial \rho }{\partial t}+\mathrm{div}\left(\rho v\right)=0,$$

or, in other words:

(14) $$\frac{d\rho }{dt}+\rho \left(\nabla \cdot v\right)=0.$$

Now, because the phase fluid is incompressible:

(15) ρ=const

on a target energy level set. Now, let us ponder ρ=C, and then, we vary the constant as C=C(x,p) to obtain the condition:

(16) ∇C·v=0→C=cH,

where *c* is a rightful constant.

The fluid should also undergo Euler’s equation (the fluid’s particle velocity denoted by v; here, we considered just the fluid of an ensemble of free harmonic oscillators with the equations of motion $\dot{x}=p,\;\dot{p}=-x$):

(17) $$\frac{dv}{dt}=-\frac{1}{\rho }\nabla P$$

which in an elementary manner leads to Bernoulli’s law, if we take the inner product of both sides with v and perform trivial integration:

(18) $$\frac{1}{2}c\left(x^{2}+p^{2}\right)+P\left(x\right)=\mathrm{const},$$

from which we can evaluate the pressure function and constant appearing in the formula, as the pressure needs to be non-negative.

## 3. M-Systems of Ecology and Chemical Kinetics

The Hamiltonian (in general Poisson) structure can be met also in ecology, where evolving populations of different species share resources in a common domain of living. Various environmental factors also can be modeled through some additional terms in differential equations governing the evolution (optimal control problems, etc. [28]).

We point out that a similar approach can be adopted for chemical kinetics problems, where the role of populations is played by the concentrations of various different chemical substances. The resource function here, if it exists, reflects the amount of reacting substances. In both domains, the systems share the properties of non-negativity, realizability, reducibility, and semi-stability [6,29,30].

The mentioned processes may involve a great number of variables, if they are to be described exactly. For our purposes, we stick to ODE models, as they are fair enough to describe phenomena with satisfying accuracy, yet simple enough not to complicate things unnecessarily, so we used the following assumptions:

1. Reagents (species) are well mixed (distributed homogeneously), otherwise the problem would be inhomogeneous in space, hence yielding Partial Differential Equations (PDEs) (e.g., the reaction–diffusion problem; see, e.g., [31]) instead of ODEs;
2. The concentrations of the substrates (species) are high enough to prevent stochastic behavior during reacting (coexisting; the abandonment of this assumption would lead to Wiener processes). Otherwise, we would obtain Stochastic Differential Equations (SDEs) (e.g., the Kubo oscillator) instead of ODEs.

Generally we could consider a few types of interaction between species: parasitic invasion, competition for resources, etc. For example, the competition of *U* and *V* given by the system:

(19) $$\begin{array}{c} \dot{u}=f\left(u,v\right),\\ \dot{v}=g\left(u,v\right) \end{array}$$

would undergo conditions $f_{u}>0,g_{v}>0,f_{v}<0,g_{u}<0$ to reflect the fact that the consumption of a nutrient/the depletion of a resource by one species would prevent the other from doing the same, as well as describing the competition between the members of the same population.

Typically considered in this context is the Lotka–Volterra system of the form:

(20) $$\begin{array}{c} \dot{u}=-\alpha u+\beta uv,\\ \dot{v}=-\gamma uv+\delta v, \end{array}$$

with a properly adjusted set of constants (note that the above equations fulfill the previously mentioned restrictions, if u,v are sufficiently bounded) and *u* being the population of the predator species and the *v*-population concentration of the prey.

A specific kind of interaction appears in systems of the Rosenzweig–MacArthur [32,33] type, involving predator and prey coexistence. In (19), we would obtain:

(21) $$\begin{array}{c} f\left(u,v\right)=ru\left(1-\frac{u}{K}\right)-vh\left(u\right),\\ g(u,v)=v(-\beta +\alpha h(u)), \end{array}$$

where h(u) denotes the number of prey caught by the predator per unit of time and α,β,r,K are the constant environmental parameters of the system.

The described model may be decomposed into symmetric and anti-symmetric part-evolutions, although it does not give up the Poissonian description at all. The ambitious objective of this paper was to consequently remedy the situation, putting (21) into not only the Poissonian, but the altogether canonical form. This type of impossibility of the direct integration of the equations of motion to solve the inverse Hamiltonian problem renders the main motivation for all efforts considered here.

Functions as h(u) are known as the functional response of the predator to a variation in the prey population. The basic types of these were classified by C.S. Holling into three categories: I. linear, II. hyperbolic (saturation), and III. so-called θ-sigmoid [33].

Various parameters in the equations of the population dynamics can be made into variable ones, often leading to their re-appearance as new dynamical (dependent) variables. To study these and more of not only single-population models such as, e.g., a chemostat system with continuous/batch cultivated bacteria, the Monod equation, the grazers–vegetation cycles, the Ivlev or Ayala–Gilpin–Ehrenfeld model of population growth, epidemic and endemic (SIR/SIS) models, or even more complicated problems associated with the optimal control of invasive species and the harvesting of populations, the reader is referred to the literature on the subject [28,34,35].

Of course, some of these systems admit Hamiltonian/Poisson representation, where we treat the resource function M(x,t) as the basic object (hence the name M-systems). It serves as a generator for the equations of motion:

(22) $$\dot{x}=B\left(x,t\right)\nabla M.$$

For instance, in the case of the Lotka-Volterra system (20) we have:

(23) $$B=\left(\begin{array}{cc} 0 & uv\\ -uv & 0 \end{array}\right)$$

with the generator:

(24) M(u,v)=−αlnv−δlnu+βv+γu

yielding the fine Poisson system or the Hamiltonian system of a non-canonical form. Sometimes, the term “M-systems” is used to describe molecular systems in biology; however, we stick to the meaning proposed in [36] and accepted by some other authors (e.g., [37]).

**Remark** **1.** *Similarly, we can formulate the Hamiltonian-like M-system substituting J in place of B and properly transforming the variables. However, a big difference occurs when we ponder both Poisson and Hamilton formulations of the same problem: the concentration variables for different species should be of non-negative values, but in the Hamiltonian case are not (incompressible fluid on $R^{m}$). Because of that, we can interpret the compressibility of the phase fluid in the Poisson case as partially arising from constraining the phase space to be $R_{+}^{m}$.*

The set of chemical reactions is termed the reaction network. Having:

(25) $$A_{i}+E_{i}\to P_{i}+B_{i},\;i=1,\ldots ,n,\;$$

we call species on the left the reactants and species on the right products of the reaction. When all stoichiometric coefficients are equal to one, we call such a reaction an elementary reaction. Note that every particular reaction can be written as an elementary reaction when we substitute $A_{1}X=X+\ldots +X$ ($A_{1}$ times). As an example, we may consider the Robertson reaction network, mentioned in Section 5, and there should be no confusion in retaining the system in the potential, Poissonian form.

Chemical reactions formulated as a population dynamics problem use the mass action law [29,35]: at constant temperature, for any elementary reaction, its rate is proportional to the concentrations of the reactants.

The matrix formulation is also accessible to problems concerning the mass action law; see, e.g., [6,29]. This simple rule underlies the differential description of such reactions as given in the Michaelis–Menten model, Hill enzymatic equation, or Robertson network.

One of the fundamental features of mass action kinetics is that it produces differential equations with polynomial non-linearities. This also means that when we encounter such a set of equations, we may find the reaction network obeying these. Such a process is referred to as the realizability of the mass action kinetics [29]. An example of this procedure may be the so-called Lotka–Volterra reactions, retrieved from (76); see [6,38].

## 4. Para-Hamiltonian Description of Non-Conservative Systems

Basically speaking, we pondered a classical system with energy $E=\frac{1}{2}p^{2}+V\left(x\right)$, where the number of dimensions is irrelevant (for now). However, in terms of Newtonian forces, in general, they are essentially non-self-adjoint (simply meaning these are not integrable [39]):

(26) $$\dot{x}=p\;,\;\dot{p}=-\mathrm{grad}\;V\left(x\right)-D\left(x,p\right)\;,$$

where *D* stands for dissipative forces.

The theory of ODEs confirms that if we are able to find a unique solution to the problem, then the phase trajectories do not intersect, in order to keep the vector field generating the ODE well defined. This means, provided sufficiently differentiable *V* and *D*, that any given instant *t* in time connects by the 1:1 correspondence to some (x,p), and vice versa. The following exposition will lean heavily upon this fact and exploit the mentioned correspondences in terms of the particular differentiation procedure along the trajectory. As an effect, we construct a new class of effectively conserved quantities. Hence, we assumed that $V\in C^{\infty },\;D\in C^{\infty }$ in all their arguments in a given domain, in a classical sense.

**Definition** **1.** *Given a Pfaff form θ=κ(x,p)dx+ν(x,p)dp (where κ and ν are some functions) and a system of equations whose trajectory (parameterized by t) maps initial data $\left(x_{0},p_{0}\right)$ to (x,p), we define:*

(27) $$\Psi \left(x,p\right)=\Psi \left(x_{0},p_{0}\right)+\int_{\gamma (t_{0},t)}\theta \;\;\equiv \;\Psi \left(x_{0},p_{0}\right)+\int_{\left(x_{0},p_{0}\right)}^{\left(x,p\right)}\left(\kappa \left(x^{\prime },p^{\prime }\right)dx^{\prime }+\nu \left(x^{\prime },p^{\prime }\right)dp^{\prime }\right),$$

*where the path of the integration is along this trajectory, denoted by $\gamma (t_{0},t)$. Then, we denote đΨ:=θ, and:*

(28) $$\frac{\partial \Psi }{\partial x(t)}:=\kappa \left(x\left(t\right),p\left(t\right)\right)\;,\;\frac{\partial \Psi }{\partial p(t)}:=\nu \left(x\left(t\right),p\left(t\right)\right)\;.$$

*Note that the above expressions are not standard partial derivatives, except in the case when the Pfaff form θ is an exact differential. In the following, these operations are referred to as derivatives along the trajectory.*

We start with the equations of motion (26) as given. Then, (assuming, for simplicity, the one-dimensional case) we seek for a “generator of motion”, however, not in a standard sense, but in the sense of Definition 1 (this is what me mean by the “para-Hamiltonian” description).

(29) $$\begin{array}{c} \dot{x}=p\overset{?}{=}\frac{\partial K}{\partial p(t)}\;\to \;K=\frac{1}{2}p^{2}+c_{1}\left(x\right),\\ \dot{p}=-V^{\prime }\left(x\right)-D\left(x,p\right)\overset{?}{=}-\frac{\partial K}{\partial x(t)}\;\to \;K=V\left(x\right)+\int_{\gamma (t_{0},t)}D\left(x,p\right)dx\;+c_{2}\left(p\right)\;, \end{array}$$

where $c_{1}\left(x\right)$ and $c_{2}\left(p\right)$ can be derived by the comparison of both equations. Hence:

(30) $$K=\frac{1}{2}p^{2}+V\left(x\right)+\int_{\gamma (t_{0},t)}D\left(x,p\right)dx$$

In other words, we define in the phase space (x,p) one-form đK, which is neither closed nor exact:

(31) $$đK=\frac{\partial K}{\partial x(t)}dx+\frac{\partial K}{\partial p(t)}dp=\left(V^{\prime }\left(x\right)+D\left(x,p\right)\right)dx+pdp\;.$$

There is ongoing energy exchange with the environment (not necessarily a loss) due to non-potential function D(x,p). We introduce a weird object *w* called the “reservoir”, dependent on *x* through the upper limit of integration. Its *x*-derivative along the trajectory is D(x,p), and theoretically, it is a redundant variable; hence:

(32) $$w=\int_{x_{0}}^{x(t)}D\left(x^{\prime }\left(t^{\prime }\right),p^{\prime }\left(t^{\prime }\left(x^{\prime }\right)\right)\right)dx^{\prime }\left(t^{\prime }\right),$$

where all needed inter-dependencies are guaranteed by the existence and uniqueness theorem for ODEs [1]. In the above formula, we perform the Riemann–Stieltjes integral to guarantee there exists some form of 1:1 correspondence between dynamical variables.

In practice, adjoining the reservoir to the system means adjoining non-potential (dissipative) forces as working for the positive account of the energy of the system (meaning they are treated as part of the system). By means of physical analysis, we incorporate the “power continuity law”:

(33) $$\frac{dE}{dt}+pD\left(x,p\right)=0\;,$$

also giving rise to one-form đK, which is perceived as a fundamental quantity of our approach (consequently, we vary only the dependent variables).

Meanwhile, it is worth noting that among the many obtained result aimed at grasping dissipative behavior correctly, the Nosé–Hoover system [13] and its generalizations [40] deserve some amount of attention. While cast in a Hamiltonian-like form (a not-so-canonical Poisson tensor), the equations of motion link the dynamics of the system to the state of the thermostat (through temperature *T*), which gives effective access to the thermodynamics of the ensemble of such systems (although not the statistical dynamics of such, the divergence of the vector field is in general non-zero). The importance of this remark rests on the similarities of this description with the one proposed here.

**Theorem** **1.** *The quantity:*

(34) K=E+w

*is conserved.*

**Proof.**  We may easily check:

(35) $$\dot{K}=\dot{E}+\dot{w}=-D\left(x,p\right)p+D\left(x,p\right)p=0;$$

however, it is not a well-defined function of *x* and *p*, since it depends on the path taken by the system. □

The above result is important not because of its complicated nature, but because of its simplicity. We accessed a novel type of conserved quantity that is rather of no use in pure theoretical considerations. As outlined in the Introduction, it is of huge practical/computational value.

Let us observe that *K* possesses an extremely trivial physical interpretation: it is just the initial energy (provided that $w(t_{0})=0$). Since, for general $\left(x_{0},p_{0}\right),w\left(t_{0}\right)=\mathrm{const}$, we have $K=E\left(x_{0},p_{0}\right)+w\left(t_{0}\right)$, therefore, *K* is a smooth function of initial values provided that *E* is smooth.

We call the differential form not being the differential of any function a (pure) Pfaffian form [41]. An example of such a quantity is:

(36) đw=D(x,p)dx.

In the future, we will denote by $w_{x}$ the expression standing next to dx in đw.

Therefore, we can define:

(37) đK=đE+đw,

as a Pfaffian differential form.

In the above definition, we paid attention to the fact that the Hamiltonian is no longer preserved. Its decrement is exactly the increment of *w* with the opposite sign. Since the change of the Hamiltonian now obviously depends on the path taken by the system on the phase space, we cannot even claim that the Hamiltonian is still a potential-type function or a properly defined function at all, although, as a shortcoming, we use the term “potential part of the generator of motion” with respect to the Hamiltonian (so, strictly speaking, dE becomes the Pfaffian form).

Since $\dot{E}=-D\left(x,p\right)p$ (see (33)), the addition of a reservoir with the exactly opposite time derivative yields the conserved quantity. Consequently, we tried to describe the problem in two different sets of canonical coordinates in several dimensions: $\left(x^{i},p_{i}\right),\left(u^{i},w_{i}\right),\;i=1,\ldots ,n$. Motion begins with $K_{0}=L_{0}=E\left(x_{0},p_{0}\right)$; energy as a number at a given time is invariant with respect to the change of coordinates (“frozen” time transformations); since the initial value $K_{0}=L_{0}$ is also invariant, it induces the invariance of the integral $w=\int_{x_{0}}^{x}D_{i}\left(x,p\right)dx^{i}$ while changing the description of the system so that:

(38) $$\begin{array}{c} đK=p_{i}dp_{i}+\nabla_{x^{i}}V\left(x\right)dx^{i}+D_{i}\left(x,p\right)dx^{i},\\ đL=w_{i}dw_{i}+\nabla_{u^{i}}\tilde{V}\left(u\right)du^{i}+F_{i}\left(u,w\right)du^{i}\;, \end{array}$$

where the summation over repeating indices is assumed.

Demanding the para-Hamiltonian framework to hold in both sets of symplectic coordinates:

(39) $$\begin{array}{cc} {\dot{x}}^{i}=\frac{\partial K}{\partial p_{i}\left(t\right)}, & {\dot{u}}^{i}=\frac{\partial L}{\partial w_{i}\left(t\right)},\\ {\dot{p}}_{i}=-\frac{\partial K}{\partial x^{i}\left(t\right)}, & {\dot{w}}_{i}=-\frac{\partial L}{\partial u^{i}\left(t\right)} \end{array}$$

we obtain the condition for action differentials to differ by an exact differential (as usual):

(40) $$p_{i}dx^{i}-w_{i}du^{i}=d\phi ,$$

where the time-dependent differentials cancel due to the common initial conditions. Traditionally performing proper Legendre transforms, we can express ϕ as a function of the preferred two subsets of the old/new variables.

Here, the integral parts with the non-potential functions match due to the invariant character of energy; the covectorial character of momenta induces $w_{j}=p_{i}\left(x,w\right)\frac{\partial p_{i}}{\partial w_{j}}$; the left-over condition gives:

(41) $$\left(D_{i}\left(x,p\right)-F_{j}\left(u,w\right))\frac{\partial u^{j}}{\partial x^{i}}\right)dx^{i}=0,$$

where we tacitly assumed $u\left(x_{0}\right)=u_{0}$. The demand for the integral to vanish is sufficient for the integrand to vanish as well. The vanishing of the integral demand is sufficient for the integrand to also vanish. However, the dependence of *D* on *p* (or *w*) forbids its form as a simple gradient function. Hence, *D* is a covector, but it is not a gradient of any scalar function.

The potential part is invariant due to the symplectic structure; hence, the dissipative form integrated from $x_{0}$ to *x* or from $u(x_{0})$ to u(x) has to be invariant in order to ensure the independence of the energy dissipated during the motion. We call such invariance secondary (or induced) invariance.

From earlier considerations, we may generalize (8) to:

(42) $$X_{K}=\pi \left(\cdot ,đK\right)$$

giving rise to the Poisson bracket:

(43) $$\left\{f,K\right\}=X_{K}\left(f\right),$$

for any function given on the phase space. We can conceive of *K* as the formal generator of non-potential motion: its Poisson bracket with canonical coordinates gives proper equations of motion:

(44) $$\begin{array}{c} \dot{x}=\left\{x,K\right\}=\pi \left(dx,đK\right)=p,\\ \dot{p}=\left\{p,K\right\}=\pi \left(dp,đK\right)=-V^{\prime }\left(x\right)-D\left(x,p\right) \end{array}$$

moreover:

(45) $$\dot{w}=\left\{w,K\right\}=\pi \left(đw,đK\right)=D\left(x,p\right)p,$$

which is indeed the case.

We can regard the condition:

(46) đK(v)=0

for any $v\in T(T^{*}M)$ as a formal substitute for the law of the conservation of energy, determining the trajectory uniquely.

In agreement with the thermodynamic description, we may think of the energy function as an analogue of the internal energy *U*. Thus, we can yield two different interpretations:

.1 Adjoining external forces to the language in which we describe the system, we obtain a closed system; hence, dU=−PdV=−đw=D(x,p)dx, and hence, the non-potential force exerts a kind of “pressure” on the ensemble of systems in the phase space (see below);
2. If đdK=dU=0, then we view TdS and PdV as equal, so reaching for the phase pressure obtained below for the phase fluid and considering phase volume dxdp as dV, we can pick the monotonically growing component of the expression PdV and treat it as an infinitesimal increment of the entropy analogue of the system, the remaining factor being the “temperature”.

Summing up all the differentiability conditions, we seek $E\left(x,p\right)\in C^{n},w\left(x\right)\in {\left(C\right)}^{1}$ such that $K=E+w\in C^{1}$, but $\nabla K\in C^{m}$, m,n arbitrary.

With this setting in mind, we can derive new formulas for the vector field algebra. Considering *K* the formal generator of motion and *f* a well-differentiable function, we obtain:

(47) $${\left[X_{f},X_{K}\right]}_{L}=-X_{\left\{f,K\right\}}+\mathrm{div}vX_{f},$$

seeing that compressible terms (v is of course a tangent vector to the phase flow, namely $X_{K}$) that are responsible for the discontinuities are causing an anomaly in the vector field algebra to occur.

It is possible to find a similar formula for the pair of reservoir-containing K,L, say. However, it is not very useful in the context of the Hamiltonian description of mechanical systems since there is only one of these needed to govern the dynamics. The situation dramatically changes in Nambu, or generalized Nambu, mechanics (e.g., [42]), where the dynamics is given in terms of a few such vector fields.

We stick to this working approach, especially since it guarantees:

(48) ω=dx∧dp

as a symplectic form. During the former considerations, it was preserved by the flow of Hamiltonian vector field $X_{H}$, and now, it satisfies:

(49) $$L_{X_{K}}\left(\omega \right)=X_{K}\rfloor d\omega +d\left(X_{K}\right\rfloor \omega )=d\left(đE+đw\right)=d\left(-đw+đw\right)=0.$$

as it provided by the equations of motion: $\dot{p}=-V^{\prime }\left(x\right)-D\left(x,p\right)$, hence $dp=-V{;}^{\prime }\left(x\right)dt-D\left(x,p\right)dt$, so multiplying both sides by *p*, we obtain $đE=pdp+V^{\prime }\left(x\right)dx=-D\left(x,p\right)dx$.

$X_{K}$ is defined unambiguously throughout the phase space as a section over $T(T^{*}M)$, hence providing that phase trajectories do not cross each other.

When it comes to the value of *K*, it is determined by providing initial conditions $\left(q_{0},p_{0}\right)$. Hence, $K\in F(T^{*}M\left(t_{0}\right))$, so its constant value is uniquely determined by the state of the system at the initial moment. As a function of the flow, *K* may be given as:

(50) $$K=\int_{t_{0}}^{t}v\rfloor \omega$$

making its dependence on the initial conditions much less manifest.

A little bit more sophisticated is the simultaneous use of two reservoirs for the system:

(51) $$\begin{array}{c} \dot{x}=p+F\left(x,p\right),\\ \dot{p}=-V^{\prime }\left(x\right)-D\left(x,p\right), \end{array}$$

yielding:

(52) $$đK=\left(p+F\left(x,p\right)\right)dp+\left(V^{\prime }\left(x\right)+D\left(x,p\right)\right)dx$$

and, accordingly:

(53) $$X_{K}=\left(p+F\left(x,p\right)\right)\frac{\partial }{\partial x}-\left(V^{\prime }\left(x\right)+D\left(x,p\right)\right)\frac{\partial }{\partial p}.$$

Since we are playing with ODE system, when we assume that $A\left(x\right)\nabla K=f\left(x\right)\in C^{r}\left(D\right),r⩾1$, the theorem on the existence and uniqueness of solutions is in power [1,19,20]. Therefore, there is a 1:1 correspondence between every moment in time and the points in the phase space. Trajectories on the phase space of the system are obviously not crossing. Hence, we can perform in an unambiguous sense any integral of a function of variables of the system with respect to some of these variables (or time) as a Riemann–Stieltjes integral.

Therefore, we can write:

(54) $$K=\frac{1}{2}p^{2}+V\left(x\right)+w+z,$$

where the reservoirs are defined by:

(55) $$\begin{array}{c} w=\int_{t_{0}}^{t}D\left(x\left(t^{\prime }\right),p\left(t^{\prime }\right)\right)dx\left(t^{\prime }\right),\\ z=\int_{t_{0}}^{t}F\left(x\left(t^{\prime }\right),p\left(t^{\prime }\right)\right)dp\left(t^{\prime }\right), \end{array}$$

so that $\dot{K}=0$.

Now, in order to make the current discussion as similar to the conservative case as possible, we focus for a moment on the hydrodynamical analogy, starting from the continuity equation:

(56) $$\frac{d\rho }{dt}+\rho \nabla \cdot v=0,$$

hence:

(57) $$\rho =Ce^{\int_{t_{0}}^{t}D_{p}\left(q,p\right)dt^{\prime }}$$

where *C* is a constant and the lower index denotes the derivative with respect to an argument.

The integral in the exponent does not cause any trouble, since all the fluid’s particles obey the equations of motion; hence, we may again apply the Riemann–Stieltjes integral.

Note that we can consider *C* as depending on the canonical variables, where from the continuity equation, we obtain the constraint v·∇C=0, so *C* may depend on the *K* value, so on the phase trajectory of interest. Here, as always, $v=X_{K}$.

Additionally, we have Bernoulli’s law (from Euler’s equation):

(58) $$\frac{1}{2}\rho v^{2}+P=\mathrm{const}.$$

Taking an example of linearly damped harmonic oscillators with the equation of motion:

(59) $$\begin{array}{c} \dot{x}=p,\\ \dot{p}=-x-bp. \end{array}$$

Since all fluid particles obey these equations of motion, the continuity equation yields:

(60) $$\rho =cKe^{bt},$$

where *c* is a constant.

Writing Bernoulli’s law:

(61) $$\frac{1}{2}cKe^{bt}\left(x^{2}+p^{2}+2bpx+b^{2}p^{2}\right)+P=\mathrm{const},$$

and remembering that $E∼e^{-bt}$, we see:

(62) $$P=P_{0}-cKe^{bt}\left(bxp+\frac{1}{2}b^{2}p^{2}\right).$$

Fortunately, we know solutions to the damped oscillator, being $x∼e^{-\frac{b}{2}t}\left(\cos\left(\omega t+\delta \right)\right)$; hence, $p∼e^{-\frac{b}{2}t}\left(\cos\left(\omega t+\delta \right)-\omega \sin\left(\omega t+\delta \right)\right)$. Thus, we that see there is no danger of the variable part of pressure growing to infinity. Provided that the engaged constants obey:

(63) $$P_{0}-cKA_{0}^{2}\left(\left(b+\frac{b^{2}}{2}\right)\cos^{2}\delta -\left(\omega b+b^{2}\right)\sin\delta \cos\delta +\frac{1}{2}b^{2}\omega^{2}\sin^{2}\delta \right)⩾0,$$

where $A_{0}$ is the initial amplitude, the pressure is always positive. Notice that for different *K* (initial energy), this demand can somewhat change quantitatively.

## 5. Particular Non-Potential Systems

Here, we give a sequence of illustrative examples, treatable along the lines of the presented approach. Their objective is to show the applicability of the invented framework to low-dimensional systems and to directly show the presence and surprising preservation of the symplectic structure. As mentioned in the Introduction, the general statement with the proof will be published elsewhere.

**Example** **1.** **van** **der** **Pol** **oscillator**  

*The van der Pol oscillator is a system that arises as some generalization of an RLC circuit (through the so-called Liénard-form equation of the VdP oscillator [19]), and its equations of motion are:*

(64) $$\begin{array}{c} \dot{x}=p,\\ \dot{p}=-x+\varepsilon \left(1-x^{2}\right)p. \end{array}$$

*The non-potential generator of motion is:*

(65) $$K=\frac{1}{2}\left(x^{2}+p^{2}\right)+w,$$

*where the reservoir variable is given by:*

(66) $$w=-\varepsilon \int_{t_{0}}^{t}\left(1-x^{2}\left(t^{\prime }\right)\right)p\left(t^{\prime }\right)dx\left(t^{\prime }\right),$$

*turning K into an effectively conserved quantity.*

**Remark** **2.** *Note that taking the ∂K(t) derivative along the phase curve, we have:*

(67) $$\left(\begin{array}{c} \dot{x}\\ \dot{p} \end{array}\right)=\left(\begin{array}{c} \;\frac{\partial K}{\partial p(t)}\\ -\frac{\partial K}{\partial x(t)} \end{array}\right)=\left(\begin{array}{cc} 0 & 1\\ -1 & 0 \end{array}\right)\nabla K$$

so that the system possesses a symplectic structure. Nevertheless, it may seem as it should not hold along the trajectory in the phase space, since $K\notin C^{2}\left(T^{*}M\right)$ (exactly, we have $K\in C^{1}\left(T^{*}M\right)$). However, due to the vanishing of đK by the construction, we have (49), and the symplectic structure is preserved.

**Remark** **3.** *Note that onward, to avoid cumbersome writing in integrals such as (66), we often write $y^{\prime }$ instead of $y(t^{\prime })$, etc.*

**Example** **2.** **Brusselator**  

*The Brusselator is the dynamical system modeling the auto-catalytic reaction network [21]:*

(68) A→X,2X+Y→3X,B+X→Y+D,X→E.

*We claim that substrates A,B are abundant in the environment, so we can denote their concentrations a,b as being constant. We identify the X and Y species’ concentrations as dynamical variables x and y, respectively. From (68), with the use of the mass action principle, we obtain:*

(69) $$\begin{array}{c} \dot{x}=a+x^{2}y-bx-x,\\ \dot{y}=bx-x^{2}y. \end{array}$$

*A quick look at these confirms there is no resource function M in the usual sense; the non-potential generator of motion becomes:*

(70) $$K=ay-\frac{1}{2}bx^{2}+w+z,$$

*where the reservoir variables are given by:*

(71) $$\begin{array}{c} w=\int_{t_{0}}^{x(t)}{\left(x^{\prime }\right)}^{2}y^{\prime }dx^{\prime }\;\\ z=\int_{t_{0}}^{y(t)}\left({\left(x^{\prime }\right)}^{2}y^{\prime }-bx^{\prime }-x^{\prime }\right)dy^{\prime }\;, \end{array}$$

*so that $\dot{K}=0$.*

**Remark** **4.** *Note that again, in the x,y variables, the system is in canonical form $\dot{x}=J\nabla K\left(t\right)$ with:*

(72) $$J=\left(\begin{array}{cc} 0 & 1\\ -1 & 0 \end{array}\right),$$

*which is preserved due to the courtesy of K being constant during the evolution.*

Note that (69) has a fixed point at $\left(a,\frac{b}{a}\right)$. This equilibrium is unstable when $b>1+a^{2}$; if $b<1+a^{2}$, it is stable. The case $b=1+a^{2}$ presents some doubts: in this situation, the origin appears to be the center (from the procedure of linearization); however, we know that if the dimension (here: the number of dependent variables) of the ODE system n⩾2, then the Hartman–Grobman theorem on linearization often fails at predicting the existence of the center [19,20,21].

**Example** **3.** **The** **Nosé–Hoover** **system** **[13,40]** *We put the thermostatted system in the form:*

(73) $$\begin{array}{c} \dot{q}=\frac{p}{m}=\frac{\partial K}{\partial p(t)},\\ \dot{p}=-\Phi_{q}-\frac{pp_{\eta }}{Q}=-\frac{\partial K}{\partial q(t)},\\ \dot{\eta }=\frac{p_{\eta }}{Q}=\frac{\partial K}{\partial p_{\eta }\left(t\right)},\\ {\dot{p}}_{\eta }=\frac{p^{2}}{m}-kT=-\frac{\partial K}{\partial \eta (t)}, \end{array}$$

*which gives:*

(74) $$K=\frac{p^{2}}{2m}+\Phi \left(q\right)+\frac{p_{\eta }^{2}}{2Q}+kT\eta +\int_{q_{0}}^{q(t)}\frac{p^{\prime }p_{\eta^{\prime }}^{\prime }}{Q}dq^{\prime }-\int_{\eta_{0}}^{\eta (t)}\frac{p^{\prime 2}}{m}d\eta^{\prime },$$

*from which, by differentiation, we can retain the equations of motion. Note that the transition to the time parameterization of the reservoirs (integral variables) shows simply K=H, the Hamiltonian presented, for example, by Ezra [13].*

**Remark** **5.** *The almost-Poisson structure provided in [40], given by:*

(75) $$B\left(x\right)=\left(\begin{array}{cccc} 0 & 0 & 1 & \frac{\tau p}{m}\\ 0 & 0 & 0 & 1\\ -1 & 0 & 0 & -p\\ -\frac{\tau p}{m} & -1 & p & 0 \end{array}\right)$$

*is obviously of the non-canonical form. The time-dimensional parameter τ governing the evolution of the Nosé variable η must be set to zero, to obtain again the simple dynamics of (73).*

*Finally, we note that in our formulation of non-potential Hamiltonian mechanics, the system is canonical with two physical degrees of freedom. This property is obviously preserved along the evolution.*

**Example** **4.** **Lotka–Volterra** **system**  

*The Lotka–Volterra model describes the basic predator–prey interaction (with a linear response):*

(76) $$\begin{array}{c} \dot{u}=u-\alpha uv,\\ \dot{v}=-\beta v+uv, \end{array}$$

*where u is the prey concentration in an environment, v is the predator concentration, α being the rate at which the consumption of the prey by a predator proceeds, and β is the death rate of a predator. Note that we chose the unit rate for the birth of the prey and the predator feeding on the prey.*

*We can write down these equations as a Poisson system:*

(77) $$\dot{x}=B\left(x\right)\nabla M,\;B\left(x\right)=\left(\begin{array}{cc} \;0 & uv\\ -uv & 0 \end{array}\right)$$

*claiming that $x={\left(u,v\right)}^{T},\nabla ={\left(\partial_{u},\partial_{v}\right)}^{T}$, and the resource function is:*

(78) M=βlnu+lnv−u−αv.

We should observe that such a formulated LV problem is given on the phase space $R_{+}^{2}$ and, as a Poisson system, has a compressible phase fluid: $\mathrm{div}\;\dot{x}=1-\beta -\alpha v+u$; compare Remark 1.

We used the Poisson structure as even-dimensional and non-degenerate, so we can bring the system to its canonical form by transformation $u=e^{q},\;v=e^{p}$, where the equations of motion become:

(79) $$\begin{array}{c} \dot{q}=1-\alpha e^{p},\\ \dot{p}=-\beta +e^{q}, \end{array}$$

with the incompressible phase fluid on the $R^{2}$ symplectic phase space with the separable resource function:

(80) $$M=p-\alpha e^{p}+\beta q-e^{q}\;.$$

Note that the LV Poisson system would become of the canonical form also with:

(81) đK=u(1−αv)dv+v(β−u)du.

Moreover, we have:

(82) $$\nabla K^{T}B\left(x\right)\nabla M=0=\nabla M^{T}J\nabla K;$$

hence, the integrals of motion commute in terms of each Poisson structure, but this only preserves the equilibria. What is more important is that we have the following.

**Corollary** **1.** *The transition from the Poisson dynamics governed by the resource function M in (78) to the canonical form, the evolution of which is dictated by (81), preserves the Poisson bracket of a target function with the generator of motion (with its coupled matrix structure).*

This remark is easily verifiable on a case-by-case basis, provided that the Poisson bracket of M with coordinates u,v is preserved.

Knowing that the non-potential generator *K* provokes the anomalies of the vector fields to occur, we expect and then obtain:

(83) $${\left[X_{f},X_{K}\right]}_{L}=-X_{\left\{f,K\right\}}+\mathrm{div}\left(X_{K}\right)X_{f}.$$

**Example** **5.** **Robertson** **reactions**  

*The reaction network:*

(84) $$X\overset{a}{\to }Y,\;Y+Y\overset{b}{\to }Y+Z,\;Y+Z\overset{c}{\to }X+Z$$

*is a system of auto-catalytic reactions where a,b,c are the reaction rates.*

*The mass action law gives a system clearly expressible in the gradient form ($x={\left(x,y,z\right)}^{T}$):*

(85) $$\dot{x}=B\left(x\right)\nabla H,\;B\left(x\right)=\left(\begin{array}{ccc} \;\;0 & cyz+by^{2} & -ax-by^{2}\\ -cyz-by^{2} & \;0 & \;ax\\ \;ax+by^{2} & \;-ax & \;0 \end{array}\right)$$

*with conserved H=x+y+z (classical rule of mass conservation). Note additionally that B(x) does not obey Jacobi’s identity, although its skew symmetry itself guarantees the conservation property (the system is almost Poisson) [12].*

*We are able to write down the system in a different form:*

(86) $$\dot{x}=\varepsilon \nabla K,$$

*ε being the totally anti-symmetric Cartesian-tensor of order three and:*

(87) $$K=-\frac{1}{2}ax^{2}-\frac{1}{3}by^{3}-a\int_{y_{0}}^{y(t)}x^{\prime }dy^{\prime }-\int_{z_{0}}^{z(t)}\left(by^{\prime 2}+cy^{\prime }z^{\prime }\right)dz^{\prime };$$

*therefore, we need a pair of reservoirs. In this form, the system is a Poisson one: the anti-symmetric structure matrix obeys the Jacobi identity; moreover, the system admits the Casimir function H, since it is obvious that ε∇H=0; hence, we can proceed with the construction of the Darboux coordinates on a single symplectic leaf of the system, e.g., (y,z), where $x=m_{0}-y-z$, $m_{0}$ constant.*

*The system reduces to:*

(88) $$\begin{array}{c} \dot{y}=\mu -ay-az-by^{2}-cyz=-K_{z},\\ \dot{z}=by^{2}=K_{y}, \end{array}$$

*where $\mu =am_{0}$. To cast the above system in the gradient form, we need only a single reservoir:*

(89) $$K=-\mu z+\frac{1}{2}az^{2}+\frac{1}{3}by^{3}+\int_{z_{0}}^{z(t)}\left(ay^{\prime }+by^{\prime 2}+cy^{\prime }z^{\prime }\right)dz^{\prime }$$

*and it is explicitly of the canonical form.*

It is worth stressing that we can apply the Casimir function to the generator governing evolution of the system (87) to reduce the number of variables, but the formula will be different from that obtained applying the given Casimir to the equations of motion and then finding the reduced generator (89). The results are obviously unequal, but their differentials are cohomologically equivalent [1].

**Example** **6.** *To illustrate that the ideas presented here work also in higher dimensions, we considered the five-dimensional system considered in [43].*

(90) $$\begin{array}{c} \dot{p}=\alpha \left(z\right)-\beta py+\gamma pq,\\ \dot{q}=\delta \left(z\right)q-\varepsilon qx-\gamma pq,\\ \dot{x}=\varepsilon qx-\zeta x,\\ \dot{y}=\beta py-\eta y,\\ \dot{z}=-\alpha \left(z\right)p-\delta \left(z\right)q+\eta y+\zeta x. \end{array}$$

*Summing all the equations together, we find:*

(91) $$\frac{d}{dt}\left(p+q+x+y+z\right)=\alpha \left(z\right)\left(1-p\right),$$

*and performing the Riemann–Stieltjes integral, we gain access to:*

(92) $$K=p+q+x+y+z-\int_{t_{0}}^{t}\alpha \left(z^{\prime }\right)\left(1-p^{\prime }\right)dt^{\prime }=C_{0}$$

*on the target level set.*

*Eliminating x, we obtain:*

(93) $$\begin{array}{c} \dot{p}=\alpha \left(z\right)-\beta py+\gamma pq,\\ \dot{q}=\delta \left(z\right)q-\varepsilon q\left(C_{0}+\int_{t_{0}}^{t}\alpha \left(z^{\prime }\right)\left(1-p^{\prime }\right)dt^{\prime }-p-q-y-z\right)-\gamma pq,\\ \dot{y}=\beta py-\eta y,\\ \dot{z}=-\alpha \left(z\right)p-\delta \left(z\right)q+\eta y+\zeta \left(C_{0}+\int_{t_{0}}^{t}\alpha \left(z^{\prime }\right)\left(1-p^{\prime }\right)dt^{\prime }-p-q-y-z\right). \end{array}$$

*which is writable in the Hamiltonian form with four reservoirs, provided we choose canonical pairs, for example (p,y),(q,z).*

Observe the interesting novelty: the traditional first integrals led to a reduction of the order of the ODE by one; effectively conserved quantities turned the problem into an integro-differential one instead. The significance of this fact and our capability to cope with it numerically will be discussed in future papers. The following example illustrates the possibility to shape the occurring systems according to our needs and taste.

**Example** **7.** *Consider the pretty general system on $R^{3}$:*

(94) $$\begin{array}{c} \dot{x}=f\left(x,y,z\right),\\ \dot{y}=g\left(x,y,z\right),\\ \dot{z}=h\left(x,y,z\right), \end{array}$$

*with given initial conditions $\left(x_{0},y_{0},z_{0}\right)$.*

*If we would like to enclose the system as Poisson, we can assume:*

(95) $$\begin{array}{c} f=aK_{y}-bK_{z},\\ g=-aK_{x}+cK_{z},\\ h=bK_{x}-cK_{y}, \end{array}$$

*with:*

(96) $$B\left(x\right)=\left(\begin{array}{ccc} 0 & a & -b\\ -a & 0 & c\\ b & -c & 0 \end{array}\right)$$

*being the Poisson structure matrix, the entries of which satisfy the Jacobi identity:*

(97) $$a_{z}-b_{y}-a_{x}+c_{z}+b_{x}-c_{y}=0.$$

*This constraint is the only requirement for a,b,c to obey; despite this, they can be truly arbitrary.*

*Solving the linear system of Equation (95), we can determine the formal generator of motion up to a constant (of course, we demand that $K_{x}$, $K_{y}$, and $K_{z}$ be sufficiently smooth functions). Doing this, we have:*

(98) $$\begin{array}{c} K_{x}=\frac{1}{2}\left(\frac{h}{b}-\frac{g}{a}\right),\\ K_{y}=\frac{1}{2}\left(\frac{f}{a}-\frac{h}{c}\right),\\ K_{z}=\frac{1}{2}\left(\frac{g}{c}-\frac{f}{b}\right). \end{array}$$

*thus introducing the maximum three reservoirs (in fact, multiplicative factors could be chosen in a much more general way).*

*Obviously, for a given B(x), we can introduce the formal Casimir function and then put the system in Hamiltonian form. Please notice that this general three-dimensional dynamical system is unique in a very specific sense: for dimensions greater than three, we obtain the whole family of non-potential generators equivalent in terms of gauge-type transformations. This remark leaves the door open for the future development of the presented reservoir approach.*

## 6. Conclusions

Our article had a twofold objective. In Section 2 and Section 3, we reintroduced some notions of the geometric language of classical mechanics in convenient notation and summarized the basic terminology of kinetic chemistry and population dynamics. The considered examples were presented in a convenient Hamiltonian-like or Poisson-like framework.

In the main section of this paper, Section 4, we chose a rather pragmatic, not to say banausic, view of the theorem of the existence and uniqueness of the solutions of ordinary differential equations and exploited this for computational ends, to gain access to the so-called non-potential (or effective) integrals of motion. Here, we emphasize that they can be treated along the lines of a weak formulation of the integrals of motion (level manifolds). As a result, we proposed a new, para-Hamiltonian, description of ODEs.

Section 5 presented versatile examples showing how to use the constructed formalism. Among a variety of formal features, our approach is distinguished by the possibility of imposing the prescribed form of the problem under study, due only to our taste and convenience, which is clearly shown in Example 7.

Apparently, the innocent play with the dependent variables provided us with a tiny bit of additional information about the dynamical system not possessing first integrals in the classical sense. Non-potential, conserved quantities are of no use from the point of view of pure mathematics; yet, they can be very helpful in the numerical analysis of dynamical problems and the construction of new algorithms. In particular, we plan to construct geometric numerical integration schemes of any order for dissipative systems, analogous to those studied in [44]. The work in this direction is in progress.

## Data Availability

Not applicable.
